# The Relationship between Injury Characteristics and Post-Traumatic Recovery after Diffuse Axonal Injury

**DOI:** 10.3390/biomedicines12020311

**Published:** 2024-01-29

**Authors:** Rita de Cássia Almeida Vieira, Leonardo Zumerkorn Pipek, Daniel Vieira de Oliveira, Wellingson Silva Paiva, Regina Marcia Cardoso de Sousa

**Affiliations:** 1 Department of Nursing, University of Sergipe, Lagarto 49400-000, Brazil; ritavieira@academico.ufs.br; 2 Nursing School, University of Sao Paulo, Sao Paulo 05508-010, Brazil; vian@usp.br; 3 Department of Neurology, Clinical Hospital of the University of Sao Paulo, University of Sao Paulo Medical School, Sao Paulo 05403-000, Brazil; 4 Department of Medicine, University of Sergipe, Lagarto 49400-000, Brazil; 5 Division of Neurosurgery, Clinical Hospital of the University of Sao Paulo, University of Sao Paulo Medical School, Sao Paulo 05403-000, Brazil; wellingsonpaiva@yahoo.com.br

**Keywords:** CT imaging, traumatic brain injury, diffuse axonal injury, functional outcomes, quality of life

## Abstract

Background: The diagnosis and prognosis of diffuse axonal injury (DAI) remain challenging. This research aimed to analyze the impact on activities of daily living (ADL), functional outcomes, quality of life (QoL), and the association between lesion severity and DAI location identified through imaging exams. Methods: This prospective cohort study included 95 patients diagnosed with DAI. Data were collected at admission, three, six, and twelve months post-injury. The associations between variables were evaluated using a mixed-effects model. Results: Functional recovery and QoL improved between three and twelve months after DAI. An interaction was observed between independence in performing ADL and subarachnoid hemorrhage (*p* = 0.043) and intraventricular hemorrhage (*p* = 0.012). Additionally, an interaction over time was observed between the Glasgow Outcome Scale (GOS) and DAI severity (*p* < 0.001), brain lesions (*p* = 0.014), and the Disability Rating Scale (DRS) with injury in brain hemispheres (*p* = 0.026) and Adams classification (*p* = 0.013). Interaction effects over time were observed with the general health perceptions and energy/vitality domains with intraventricular hemorrhage, and the social functioning domain with the obliteration of basal cisterns and Gentry’s classification. Conclusion: The use of CT in the acute phase of DAI is important for predicting outcomes. The severity and location of DAI are associated with functional outcomes, ADL, and QoL.

## 1. Introduction

Diffuse axonal injury (DAI) is a microscopic injury that affects the axons in the cerebral hemispheres, corpus callosum, and brainstem. DAI is clinically characterized by a coma lasting six hours or more following a traumatic brain injury (TBI), excluding cases of expanding or ischemic brain injuries [1]. The cognitive, physical, and behavioral changes caused by DAI compromise in activities of daily living (ADL) and quality of life (QoL). These consequences extend beyond the acute phase of treatment, persisting and evolving over an extended period after the traumatic event. During recovery, the brain can gradually regain some function through the neural connection remodeling facilitated by brain plasticity [2,3,4,5].

The severity and outcome of DAI are influenced by the location and number of DAI lesions [6,7,8,9]. Imaging exams have been used to identify these injuries and estimate outcomes, but the prognostic value of DAI characteristics is not well established [6,7,8,9]. Mata-Mbemba et al. [10] have shown that an intraventricular hemorrhage identified in initial computerized tomography (CT) is a marker of DAI, specifically moderate and severe DAI [10]. Chelly et al. [6] observed that the classification of DAI according to Gentry’s classification [11], based on the location and extent of the injury, does not have a prognostic value even in patients with isolated DAI. In his cohort, the faster recovery of consciousness and lesions in the corpus callosum were correlated with a better prognosis after 12 months [6]. Similarly, Kim et al. [12] showed that there is no statistically significant difference between the clinical outcomes between patients with pure or non-pure DAI. In these studies, Magnetic Resonance Imaging (MRI) has been used to evaluate the extent and distribution of DAI, but the prognostic value of the characteristics of this lesion has not been well established.

Currently, MRI is typically used in the later stages of trauma treatment to assess brain atrophy and to confirm the diagnosis of DAI when CT does not show any brain injury lesion or small hemorrhage [13]. However, a study that compared the results of the MRI exams performed in patients of DAI showed that only early MRI findings helped to predict clinical outcomes [7]. Therefore, an early evaluation with CT may be more valuable for estimating prognosis in clinical practice than MRI, which is not usually available or only happens later. In middle- and low-income countries, where access to MRI may be limited, the use of CT in the acute phase of trauma to initially identify the injury can be useful to estimate patient prognosis and predict long-term recovery. The presence of an intraventricular hemorrhage, subarachnoid hemorrhage, and gliding contusion are indirect signs of DAI that may be present on CT [2].

This study aims to describe the recovery of patients with DAI between discharge and one year post-injury, focusing on functional outcomes, ADL, Instrumental Activities of Daily Living Scale (IADL), and QoL. Additionally, we aim to analyze the association between DAI severity and location on acute CT and the post-traumatic consequences at three, six, and twelve months after DAI.

## 2. Materials and Methods

This is a prospective cohort study with four time points for data collection: during hospitalization, discharge, three, six, and twelve months after trauma. The study was conducted at a specialized trauma hospital in the state of Sao Paulo, Brazil. The study included patients diagnosed with DAI who were hospitalized from July 2016 to December 2018. This project was approved by the Ethics and Research Committee of the Hospital da Clinicas da Faculdade de Medicina de São Paulo, under number 1.645.348, CAEE 51614015.6.3001.0068. Patients or their responsible family members provided verbal and written consent to participate in the study by signing the informed consent form.

Patients were selected if they had a Glasgow Coma Scale (GCS) score of ≤8 upon hospital admission after proper systemic reanimation and a diagnosis of DAI (without any combined focal injury), were admitted directly from the trauma scene, and were between 18 and 60 years old. Patients with focal or mass effect injuries (e.g., subdural hematoma, epidural hematoma, contusion) were also excluded from the study, even if these injuries were associated with the presence of DAI on CT and/or MRI.

The study excluded individuals who were treated at this hospital more than six hours after the traumatic event, those transferred from other hospitals, those with a previous diagnosis of traumatic brain injury (TBI), psychiatric disorders, or other head and spinal cord injuries of severity ≥ 3 according to the Abbreviated Injury Scale (AIS) [14]. Patients with AIS ≥ 3 injuries in specific body regions may experience long-term deficiencies and disabilities after trauma [15], making it difficult to distinguish the consequences of DAI in the recovery process.

**Figure 1:**
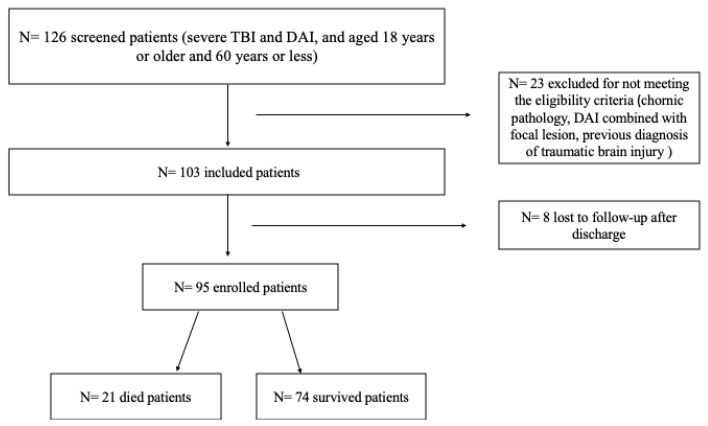
Patient recruitment flow chart.

A total of 103 patients met the inclusion criteria and were included in the study. Out of the 126 patients admitted to the hospital with TBI between August 2016 to December 2018, 23 were excluded for not meeting the eligibility criteria. Of the 103 patients included, 8 (8.4%) were lost to follow-up after discharge, leaving 95 patients for analysis in the follow-up study (Figure 1).

### 2.1. Measurements

The following sociodemographic and clinical variables were obtained: gender, age, education, marital status, race, occupational status, GCS, presence of orotracheal intubation (OTI), pupillary changes, use of continuous sedation, duration of continuous sedation, duration of coma, severity of DAI, length of stay, Maximum Abbreviated Injury Scale (MAIS) [14], Injury Severity Score (ISS) [15], New Injury Severity Score (NISS) [16], The Corticosteroid Randomization After Significant Head Injury (CRASH) CT mortality, and unfavorable outcome at six months.

### 2.2. Post-Traumatic Brain Recovery Outcomes

Functional Recovery was evaluated using the Glasgow Outcome Scale (GOS) and Disability Rating Scale (DRS), which aim to quantify functional capacity after brain injury [17]. The GOS score presents eight categories, ranging from zero (total recovery) to seven (death), while the DRS classifies patients into ten categories, ranging from zero (without disability) to 30 (death) [18,19]. In addition to functional capacity three to twelve months after DAI, independence in activities of daily living (ADLs) and instrumental activities of daily living (IADLs) were evaluated. Performance in ADLs was evaluated using the Katz scale, which measures independence in performing activities such as bathing, dressing, personal care, transfers, toilet use, and continence. Scores range from 0 to 18, with lower scores indicating greater independence [20]. IADLs were evaluated using the Lawton scale, which measures the ability to perform more complex activities such as managing finances, using the telephone, shopping, food preparation, housekeeping, laundry, mode of transportation, responsibility for own medication, and the ability to handle finances. The score ranges from 8 to 24, with higher scores indicating greater independence [21].

Quality of life was evaluated using the Short Form 36 (SF-36), which measures eight domains of a health-related quality of life represented by 36 questions: physical functioning; role limitations (physical); bodily pain; general health perceptions; energy/vitality; social functioning; role limitations (emotional); and mental health [22]. After recalibrating two items and reversing the score of 9 items, the responses to items are summed. Scores range from 0 to 100, with 0 indicating the least favorable quality of life (QoL) status and 100 indicating the most favorable QoL.

Imaging exams (CT and MRI) were used to identify the presence and location of DAI by radiologists and neurosurgeons. The presence of an intraventricular hemorrhage and the obliteration of basal cisterns were identified through imaging exams. The severity and location of DAI were evaluated using the Marshall CT Score, Adams classification, and Gennarelli’s. The Marshall CT score is classified as follows: Diffuse Injury I includes all the injuries where there is no visible pathology; Diffuse Injury II includes all diffuse injuries in which the cisterns are present, the midline shift is less than 5 mm, and/or there is no high- or mixed-density lesion of more than 25 cc; Diffuse Injury III includes diffuse injuries with swelling where the cisterns are compressed or absent and the midline shift is 0 to 5 mm with no high- or mixed-density lesion of more than 25 cc; and Diffuse Injury IV includes diffuse injuries with a midline shift of more than 5 mm and no high- or mixed-density lesion of more than 25 cc [23]. Adams classification defines grade 1 as evidence of axonal damage in the white matter of the cerebral hemispheres, including the corpus callosum, brainstem, and occasionally the cerebellum; grade 2 includes focal lesions in the corpus callosum in addition to diffuse axonal damage; and grade 3 includes focal lesions in both the corpus callosum and dorsolateral quadrant of the rostral brainstem [11]. Gennarelli defined the severity of DAI clinically: mild DAI with a coma duration of 6–24 h; moderate DAI with a coma duration > 24 h without brainstem involvement; and severe DAI with a coma duration > 24 h with brain signs [1].

### 2.3. Data Collection

Data were collected during hospitalization, at discharge, and three, six, and twelve months after DAI. Sociodemographic and clinical variables were collected upon admission and during hospitalization. Functional capacity, independence for ADLs and IADLs, and QoL were evaluated at discharge, three, six, and twelve months after DAI. Patient follow-up was conducted at the specialty outpatient clinic. All patients were instructed, upon hospital discharge, to attend the outpatient clinic for follow-up appointments at three, six, and twelve months after DAI. Confirmations and appointment scheduling were carried out through a previous telephone contact. Patients who remained hospitalized at three, six, or twelve months after DAI were assessed during the follow-up period. The CT imaging exams used to identify the presence and location of DAI were performed upon admission and repeated within 48 h after admission, while MRI exams were performed until discharge or during the follow-up period (Supplement Figure S1).

### 2.4. Statistical Analysis

Descriptive statistics were used to summarize the characteristics of the sample, including sociodemographic and clinical variables. Categorical variables were compared using the chi-square test or Fisher’s exact test, while continuous variables were compared using the student’s *t*-test or analysis of variance (ANOVA). To assess the associations between the presence and location that DAI identified on imaging exams and the outcomes of functional capacity, independence for ADLs and IADLs, and QoL, the chi-square test and Fisher’s exact test were used. Furthermore, the associations between the severity and location of DAI and the outcomes (functional capacity, independence for ADLs and IADLs, and QoL) were evaluated using a mixed-effects model.

A *p*-value of less than 0.05 was considered statistically significant, indicating a significant association between variables. All statistical analyses were performed using the R version 4.1.3 software program.

## 3. Results

At admission, all patients (n = 95) were in a coma with a GCS score of 8 or less. The average GCS score was 4.7 (SD = 1.8), the pupillary GCS was 4.3 (SD = 2.0), and the average Richmond Agitation-Sedation Scale (RASS) score was −4.6 (SD = 0.7). The majority of patients were intubated (74.7%) and sedated (85.3%). Bilateral pupils’ reactivity was present in 73 patients (76.8%), while 16 patients (16.8%) had both pupils non-reactive.

The evaluation of trauma severity showed an average ISS of 34.1 (SD = 11.2), NISS of 43.5 (SD = 14.6), and head AIS of 4.6 (SD = 0.5). The mean probabilities for a 14-day mortality and 6-month unfavorable outcome according to the CRASH CT were 36.96% (SD = 18.7%) and 71.7% (SD = 16.8%), respectively. Table 1 presents the findings of the CT scans, where it was observed that the majority of patients had a normal CT (26.3%). Approximately half of the participants presented early signs of TBI, such as subarachnoid hemorrhage (SAH) (48.4%) and gladding contusion (45.3%).

The average sedation time was 4.3 days (SD = 5.6 days), and the average duration of coma was 12.7 days (SD = 41.1 days). Mild TBI was observed in 45.3% of patients, moderate TBI in 27.4%, and severe TBI in 27.4%. Almost all cases were admitted to the ICU (91.6%) and had continuous sedation (93.7%). The average length of stay in the ICU was 16.8 days (SD = 40.9 days), and the average hospital stay was 19.8 days (SD = 22.46).

### 3.1. Recovery of Surving Patients

The frequency of patients categorized in different categories of the GOS, DRS, Katz Index, and Lawton scales is presented in Table 2. At the twelve-month follow-up after DAI, 74 (77.9%) patients had survived while 21 (22.1%) patients had died. Over time, there was a decrease in the frequency of individuals unable to recover, accompanied by an increase in those categorized as being in good recovery. The non-parametric Friedman test revealed a significant difference in the categorizations of the GOS, Katz Index, and DRS between the evaluation moments—discharge, 3, 6, and 12 months—with a *p*-value of less than 0.001.

**Figure 2:**
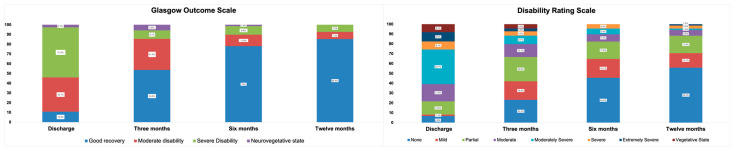
Stratification for Glasgow Outcome Scale and Disability Rating Scale at discharge, three months, six months, and twelve months after diffuse axonal injury (n = 74 surviving patients).

Patients with DAI (n = 74) demonstrated functional improvement during the initial six months following the injury (Figure 2). During the follow-up, it was observed that, at three and six months, 34 out of the 74 surviving patients underwent some form of treatment with rehabilitation specialists (other medical specialty, physiotherapists, occupational therapists, speech therapists). However, by twelve months, this number decreased to 26 out of the 74 surviving patients. It is important to highlight that only one patient remained hospitalized for six days during rehabilitation.

### 3.2. Quality of Life (QoL)

In the descriptive analysis of the eight domains of (QoL) assessed by the SF-36, it was observed that the social and mental health domains had the highest QoL scores over time, while the physical and emotional limitation domains had the lowest QoL scores (Table 3).

### 3.3. Imaging and Functional Recovery

**Figure 3:**
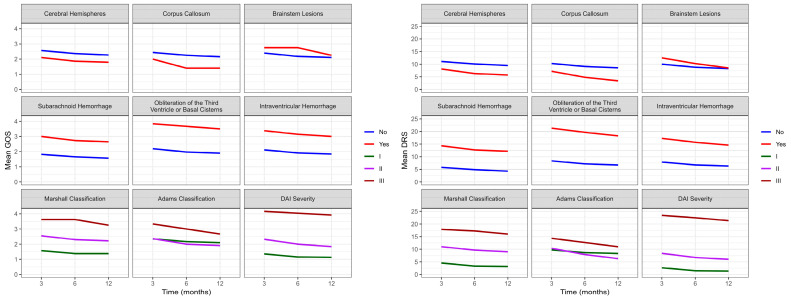
Evolution of the average functional recovery assessed by GOS and DRS in DAI patients (n = 74) at three, six, and twelve months after trauma. GOS: Glasgow Outcome Scale; DRS: Disability Rating Scale; DAI: diffuse axonal injury.

**Figure 4:**
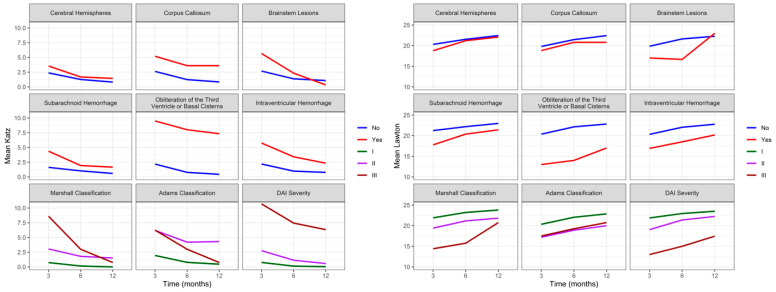
Evolution of independence to perform ADLs and IADL assessed by Katz and Lawton in DAI patients (n = 74) at three, six, and twelve months after trauma. ADLs: activities of daily living; IADL: instrumental activities of daily living; DAI: diffuse axonal injury.

The analysis of functional recovery and injury location showed that patients with injuries in the brain hemispheres (75.9% at all time points), corpus callosum (60.0% at three months; 80.0% at six and twelve months), and brain stem (42.8% at three and six months; 71.4% at twelve months) scored above 70.0% for favorable outcomes (good or moderate recovery) at twelve months, with a more pronounced improvement in favorable outcomes in individuals with injuries located in the brain stem. By 12 months after DAI, 13.8% of patients with injuries located in the brain hemispheres and 28.6% with injuries in the brain stem died. No patients with injuries in the corpus callosum died during the evaluation period. Figure 3 and Figure 4 show evidence of the interaction effect of the GOS, DRS, Katz, and Lawton over time in DAI patients (*p* < 0.05).

Figure 3 shows evidence of the main effect for cerebral hemispheres (*p* = 0.036), brain lesions (*p* = 0.015), and DAI severity (*p* < 0.001) with GOS. However, no interaction was observed between functionality evaluated by GOS over time (three, six, and twelve months) and injury location or severity. The mixed effects model analysis showed significant differences between cerebral hemispheres (*p* = 0.028), brainstem lesions (*p* = 0.014), Adams classification (0.049), and DAI severity (*p* < 0.001) with DRS. Additionally, there was an interaction between functionality evaluated by DRS over time and cerebral hemispheres (*p* = 0.026) and Adams classification (*p* = 0.013). The worst scores on DRS were observed in Adams classification II and III; however, it was observed that patients with classification II had a pronounced improvement over time, achieving a more pronounced functional improvement than patients with Adams classification I.

Figure 4 shows a correlation between Katz and brain lesions (*p* = 0.001), the obliteration of the third ventricle or basal cisterns (*p* = 0.016), an intraventricular hemorrhage (*p* = 0.029), and the severity of DAI (*p* < 0.001). Over time, we can observe evidence of an effect and interaction between Katz and HSA (*p* = 0.043) and an intraventricular hemorrhage (*p* = 0.012). Although we observe the worst Katz scores at three months, patients with an intraventricular hemorrhage showed a significant improvement over time, not reaching the same level of independence as patients without this injury. There is evidence of interaction effects from brain lesions (*p* = 0.002), the obliteration of the third ventricle or basal cisterns (*p* = 0.008), an intraventricular hemorrhage (*p* = 0.001), Marshall Classification (*p* = 0.012), and the severity of DAI (*p* < 0.001) with IADL. However, the mixed effects model did not show evidence of an interaction effect between Lawton and the location or severity of the injury over time (three, six, and twelve months).

### 3.4. Imaging and Quality of Life

**Figure 5:**
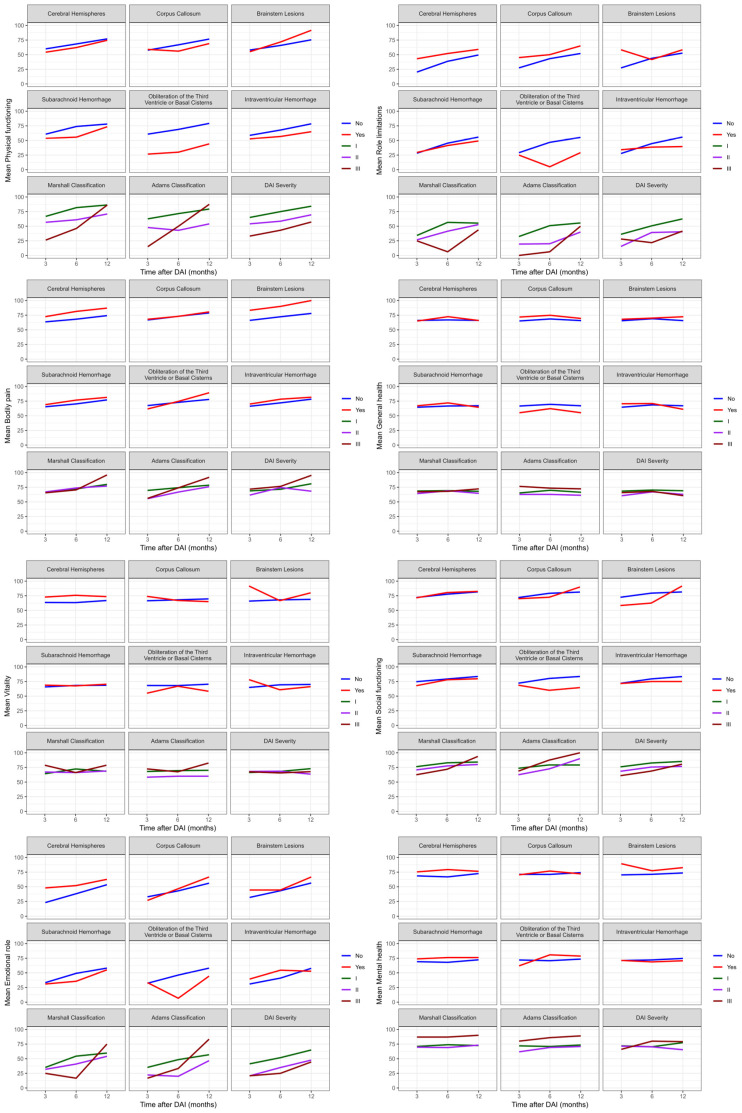
Evolution of quality of life assessed by SF-36 in DAI patients at three, six, and twelve months after trauma. SF-36: Short Form-36; DAI: diffuse axonal injury.

Mixed effects model analysis showed that there were significant differences (*p* < 0.05) between role limitations (emotional) and brain lesions (*p* = 0.025), energy/vitality with brain hemisphere lesions (*p* = 0.037), and the physical functioning and obliteration of basal cisterns (*p* = 0.029). Figure 5 showed evidence of interaction effects over time with the General health perceptions (*p* = 0.018) and energy/vitality (*p* = 0.024) domains when an intraventricular hemorrhage was present. This means that patients with an intraventricular hemorrhage experienced a worsening perception of their health, energy levels, and vitality after six months of DAI, with a slight improvement in perception in the following months.

Additionally, the social functioning domain showed interaction effects over the twelve months with the obliteration of basal cisterns (*p* = 0.046) and Adams classification (*p* = 0.020), as assessed by the SF-36. Therefore, DAI patients experienced a reduction in patient integration in social activities after the first six months of trauma, while patients with Adams classification II and III showed improvement in this interaction over time. The other domains did not show interactions over time with the location and severity of DAI.

## 4. Discussion

This study evaluated functional recovery, QoL, and independence in performing ADL and IADL in patients with DAI within one year after trauma. Similar to previous studies [24,25,26], our findings showed that patients demonstrated a good evolution in functional recovery and independence over time. Henninger et al. [27] showed that patients with predominantly hemorrhagic DAI had a significant improvement between three (52%) and twelve months (75%) after injury. A systematic review showed that 52% of patients with DAI had a favorable outcome after a mean follow-up of nine months [25]. Moreover, patients with DAI had an odds ratio (OR) of 2.9 (95% CI 1.4–6.0) for an unfavorable outcome when compared with TBI patients without DAI [25]. In our study, at twelve months after DAI, 61.1% of patients had complete recovery according to the GOS, and 50.5% had mild disability or no disability.

After DAI, changes in functional capacity may compromise individuals’ ability to perform basic ADL, such as bathing, eating, and dressing, or IADL, which involve more complex tasks than ADL. A previous study conducted with 110 patients with consciousness disorders after moderate and severe TBI showed independence in carrying out self-care activities during the first five years after the injury [28]. A study conducted with 131 adults after severe TBI with focal lesions showed an improvement in independence to perform ADL and IADL over time, where approximately 79% of patients had independence in performing ADL at twelve months, while more than half, 54%, were independent in performing more complex activities such as managing finances, using the telephone, shopping, food preparation, and housekeeping [29]. In our study, at twelve months after DAI, 46.3% of patients had the maximum score on the Lawton Scale, indicating complete independence in performing IADL.

It is known that these functional limitations and difficulties in ADL can affect the QoL of patients after trauma, especially in severely affected patients. A study demonstrated a strong correlation between QoL and functionality, indicating that patients with high QoL scores, with a better perception of their quality of life, showed more favorable results in terms of functionality [25]. Our findings showed that all patients reported an improvement in QoL after DAI in most domains, except for the general health domain, which showed a decline from six to twelve months after the injury. Furthermore, the role limitations (emotional and physical) domains had the lowest scores throughout the evaluation period in our study, while the social functioning and mental health domains had the highest QoL values. Van Eijck et al. [30] showed that out of 185 patients diagnosed with DAI in the Netherlands, where 86 were evaluated for QoL, 62% reported a good quality of life (scores between 60 and 100%) after a median time of 54 months (range 14–100 months) assessed by the Quality of Life After Brain Injury (QOLIBRI) scale [30]. In contrast to our findings, these authors showed that the emotional (median of 80%, range 15–100%), physical problems (median of 75%, range 10–100%), daily life/autonomy (median of 71.4%, range 3.6–100%), and social relationships (median of 70.8%, range 12.5–100%) domains had higher QoL scores.

Diagnosing DAI on imaging is a common challenge in many healthcare services. CT scans can show signs of SAH and an intraparenchymal hemorrhage [10,24,25,26]. Although studies show that MRI can detect intraparenchymal lesions up to three times more accurately, its feasibility in the acute phase of trauma is hindered by the high cost, long execution time, and physiological instability of patients with severe TBI who require continuous monitoring. Moreover, these studies only include DAI survivors, which limits its application for outcome prediction in clinical practice [10,24,25,26]. In a systematic review, Vieira et al. [24] showed that the most frequent lesions on CT scans in patients with DAI were SAH, intraventricular hemorrhage, blood in cisterns, and hemorrhagic DAI. The presence of intraventricular hemorrhage in the initial CT scan can also be a marker for severe DAI, but only when the CT scan identifies midline SAH [24]. Consistent with previous publications, our study shows that the most frequent lesions on imaging studies were SAH (48.4%), contusion (45.3%), intraventricular hemorrhage (23.2%), and lesions located in the cerebral hemispheres (30.5%).

Studies often use lesion location and imaging tests to predict the severity of DAI in patients. In our study, we observed an interaction between Adams classification over time and functionality assessed by the DRS. Interestingly, a narrative review published in 2021 found that Adams “Grade III” classification does not have a proven association with outcomes after traumatic DAI [31]. It is important to note that in the acute phase of severe TBI, patients may not be in a clinical condition to undergo MRI, and CT is the preferred imaging modality for identifying the lesion in the acute phase of trauma. Additionally, in low- and middle-income countries, access to MRI exams is not readily available, especially after hospital discharge where it can take years to obtain access. It is known that the Gentry classification is frequently used in MRI reports, which is an adaptation of the Adams classification [32]. Although Gentry classification does not reflect the severity of the injury, its application in patients with DAI is important for lesion localization and its association with the outcome [31,33]. Van Eijck, in a systematic review and meta-analysis published in 2018, identified that the most common lesions observed on MRI in patients with DAI are in the corpus callosum, brainstem, and thalamus, with only lesions in the corpus callosum consistently reported to be related to outcome [25].

Our findings showed an interaction over time between an intraventricular hemorrhage and ADL. Mata-Mbemba et al. [10]. conducted a study with 140 TBI patients in Japan, showing that an intraventricular hemorrhage was an independently associated predictor of DAI (OR 4.2, 95% CI = 1.3–14.3), with a positive correlation with Gentry III classification. However, these authors did not demonstrate the relationship between the presence of an intraventricular hemorrhage and independence in performing ADL [10]. In addition to functionality and independence for ADL, our study also evaluated the interaction of the eight QoL domains at three, six, and twelve months with the severity, type, and location of injury. In this context, an interaction was observed between the general health perceptions and energy/vitality domains with an intraventricular hemorrhage, and the social functioning domain with the obliteration of basal cisterns and Gentry’s classification.

In contrast to our findings, Eijck et al. [30] showed that Marshall’s classification, Gentry’s classification, and the return of consciousness after seven days were not independent factors associated with quality of life in DAI patients, as assessed by the QOLIBRI questionnaire. In the same study, the authors demonstrated that patients with an unfavorable outcome on the GOS also scored low on the QOLIBRI, indicating a poor quality of life. Patients with DAI 3 had a median QOLIBRI score of 58.7 (range 16–99), which was lower than patients with DAI 1 (median 69.4; range 23.6–100) and DAI 2 (median 71; range 34.7–94) [30]. Despite studies highlighting the importance of identifying the pattern of functional recovery and quality of life after severe TBI, few studies have evaluated the relationship between quality of life, independence in performing ADLs and IADLs, severity, and the location of the lesion after DAI and severe TBI.

It is important to identify the functional recovery pattern, including independence for ADLs and quality of life, after DAI, as this evaluation is often overlooked in these patients. Our study is the first to examine the interaction of these outcomes over time in a longitudinal manner. Furthermore, the use of CT findings and the duration of the coma as possible indications of DAI is extremely relevant for predicting outcomes and implementing clinical practices in real-life settings, where access to MRI exams is limited and usually delayed for survivors. Thus, our findings indicate that the application of CT in the acute phase of DAI is an important resource to be implemented in clinical practice to predict the outcomes and consequences of DAI patients in low- to middle-income countries, where the majority of TBI patients do not have the opportunity to undergo MRI.

There are some limitations that need to be highlighted. Only a portion of our cohort underwent an MRI during the twelve-month follow-up (30%), but in all of them had findings compatible with DAI. As most of the exams were performed after hospital discharge, the inability to schedule them due to high internal demand made it impossible to perform these exams in all patients. Additionally, the absence of hospital records on the patient’s clinical condition at the scene of the trauma and during transport limits the identification of factors in the prehospital phase associated with the outcomes of DAI, especially the GCS before intubation. The high adherence of patients to follow-up consultations up to twelve months was reinforced by daily contact with patients and their families during hospitalization, as well as frequent calls to schedule consultations, which helped to maintain trust between patients and the team during the follow-up period.

## 5. Conclusions

Our study provides valuable insights into the functional recovery, ADL, IADL, and QoL of patients with DAI. Our findings suggest that the severity and location of DAI are important predictors of long-term outcomes. Specifically, we observed an improvement in functional recovery, ADL, IADL, and QoL within the first six months after injury, but a decrease in the General health perceptions domain of the SF-36 from six to twelve months. Additionally, the role limitations (emotional and physical) domains had the lowest scores throughout the evaluation period in our study, while the social functioning and mental health domains had the highest QoL values.

Importantly, we identified the interactions over time between different levels of severity and the mean Katz and GOS scores. The independence to perform ADL showed interaction over time with an intraventricular hemorrhage, while functionality showed an interaction with Adams classification. Furthermore, in the evaluation of the eight QoL domains by the SF-36, we found an interaction between the general health perceptions and energy/vitality domains with an intraventricular hemorrhage, and the social functioning domain with the obliteration of basal cisterns and Gentry’s classification.

Our study highlights the importance of early diagnosis and targeted interventions to improve the functional recovery and QoL of patients with DAI. It also emphasizes the utility of CT in the acute phase of DAI for predicting outcomes, particularly in low- to middle-income countries where access to MRI exams is limited. The findings underscore the need for long-term follow-up and multidisciplinary care to optimize the recovery and well-being of patients with DAI. Further research is warranted to better understand the mechanisms underlying the association between DAI characteristics and outcomes, and to develop more effective interventions for improving the long-term outcomes of patients with DAI.

## Figures and Tables

**Table 1:** Injury identification and clinical characteristics of DAI patients (n = 95) during hospitalization and follow-up imaging exams.

Variables	n^o^ (%)
Marshall CT score	
Type I	25 (26.3)
Type II	61 (64.2)
Type III	9 (9.5)
Adams classification	
I	76 (80.0)
II	13 (13.7)
III	6 (6.3)
Subarachnoid hemorrhage	
Yes	46 (48.4)
No	49 (51.6)
Intraventricular hemorrhage	
Yes	22 (23.2)
No	73 (76.8)
Brainstem hemorrhage	
Yes	4 (4.2)
No	91 (95.8)
Gliding contusion	
Yes	43 (45.3)
No	52 (54.7)
Diffuse swelling	
Yes	4 (4.2)
No	91 (95.8)
Corpus callosum	
Yes	5 (5.3)
No	90 (94.7)
Cerebral hemispheres	
Yes	29 (30.5)
No	66 (69.5)
Brainstem	
Yes	7 (7.4)
No	88 (92.6)
Intracranial hypertension within 48 h of DAI	
Yes	10 (10.5)
No	85 (89.5)
DAI severity	
Mild	43 (45.3)
Moderate	26 (27.4)
Severe	26 (27.4)

CT: computerized tomography; DAI: diffuse axonal injury.

**Table 2:** Comparison of scores of GOS, DRS, Katz Index scales between discharge, three, six, and twelve months and Lawton between three, six, and twelve months after DAI (n = 74 surviving patients).

Scale	Timing	Average (SD)	*p*-Value
GOS	Discharge	3.0 (1.2)	<0.001
	3 months	2.4 (1.6)	
	6 months	2.2 (1.7)	
	12 months	2.1 (1.7)	
DRS	Discharge	13.6 (10.5)	<0.001
	3 months	10.1 (12.0)	
	6 months	8.8 (12.4)	
	12 months	8.3 (12.5)	
KATZ	Discharge	7.86 (5.9)	<0.001
	3 months	2.8 (5.2)	
	6 months	1.4 (3.8)	
	12 months	1.0 (3.5)	
Lawton	3 months	19.7 (4.9)	<0.001
	6 months	21.4 (4.3)	
	12 months	22.31 (3.8)	

GOS: Glasgow Outcome Scale; DRS: Disability Rating Scale; DAI: diffuse axonal injury; SD—standard deviation.

**Table 3:** Comparison of SF-36 domain scores between three, six, and twelve months after DAI.

Domains	Average (SD)	*p* Value
General health perceptions		
3 months	65.6 (16.7)	<0.001
6 months	69.0 (17.6)	
12 months	66.0 (17.5)	
Physical functioning		
3 months	57.8 (33.3)	<0.001
6 months	66.0 (30.7)	
12 months	76.1 (30.0)	
Role limitations (physical)		
3 months	28.7 (38.5)	<0.001
6 months	43.7 (47.1)	
12 months	52.9 (43.9)	
Bodily pain		
3 months	66.9 (25.0)	<0.001
6 months	73.1 (26.4)	
12 months	79.0 (26.1)	
Energy/vitality		
3 months	67.0 (22.4)	<0.001
6 months	68.0 (22.0)	
12 months	69.3 (23.0)	
Social functioning		
3 months	71.8 (29.4)	<0.001
6 months	78.7 (27.5)	
12 months	82.0 (27.5)	
Role limitations (emotional)		
3 months	32.3 (42.2)	<0.001
6 months	43.3 (43.8)	
12 months	56.9 (43.1)	
Mental health		
3 months	71.1 (22.9)	<0.001
6 months	71.5 (24.0)	
12 months	74.0 (24.5)	

SD: standard deviation; SF-36: Short Form-36; DAI: diffuse axonal injury.

## Data Availability

All the data are contained in the manuscript.

## Supplementary Materials

The following supporting information can be downloaded at: https://www.mdpi.com/article/10.3390/biomedicines12020311/s1, Figure S1: Data collection timeline.
