# Supplementary figures and images for: The Relationship between Injury Characteristics and Post-Traumatic Recovery after Diffuse Axonal Injury
Source: Biomedicines. 2024 Jan 29;12(2):311. doi: 10.3390/biomedicines12020311 (PMC10886783; doi:10.3390/biomedicines12020311)

Supplement Figure S1: Data collection timeline

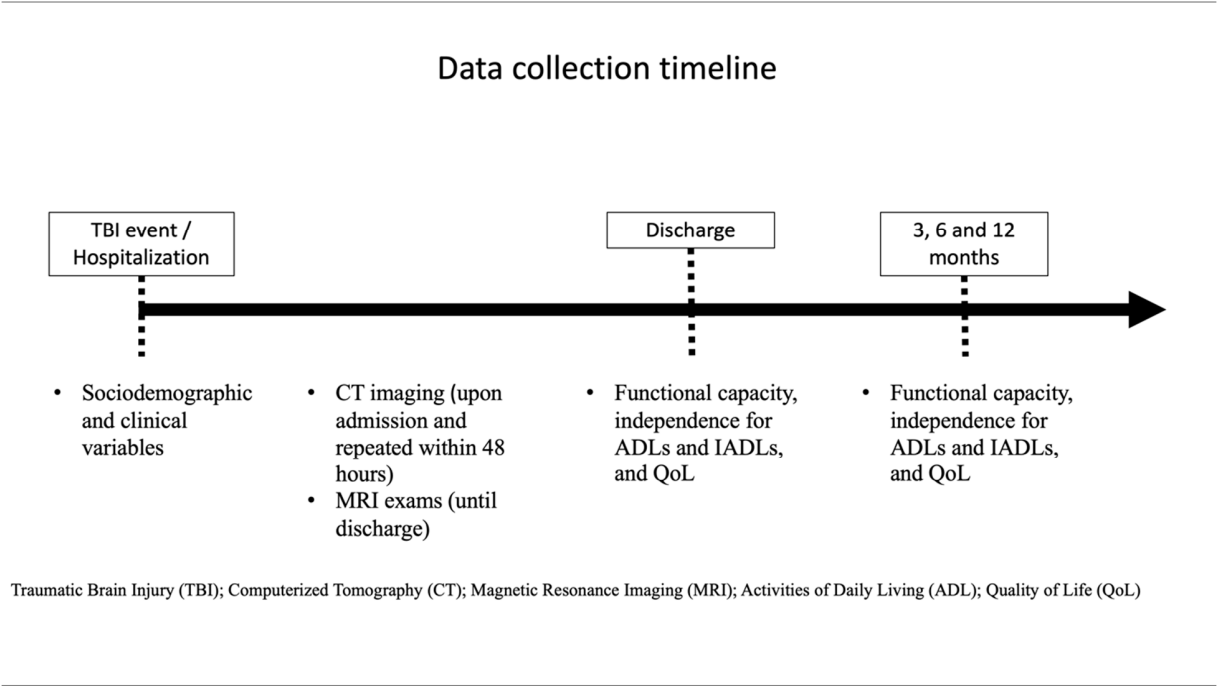

Supplement: Supplementary file 1 [file biomedicines-12-00311-s001.zip › biomedicines-2779212-supplementary.pdf]
